# At the frontiers of surgery: review

**DOI:** 10.1186/1758-3284-3-7

**Published:** 2011-02-09

**Authors:** Tahwinder Upile, Waseem K Jerjes, Henricus J Sterenborg, Brian J Wong, Adel K El-Naggar, Justus F Ilgner, Ann Sandison, Max J Witjes, Merrill A Biel, Robert van Veen, Zaid Hamdoon, Ann Gillenwater, Charles A Mosse, Dominic J Robinson, Christian S Betz, Herbert Stepp, Lina Bolotine, Gordon McKenzie, Hugh Barr, Zhongping Chen, Kristian Berg, Anil K D'Cruz, Holger Sudhoff, Nicholas Stone, Catherine Kendall, Sheila Fisher, Alexander J MacRobert, Andreas Leunig, Malini Olivo, Rebecca Richards-Kortum, Khee C Soo, Vanderlei Bagnato, Lin-Ping Choo-Smith, Katarina Svanberg, I Bing Tan, Brian C Wilson, Herbert Wolfsen, Irving Bigio, Arjun G Yodh, Colin Hopper

**Affiliations:** 1 The "Head and Neck Optical Diagnostics Society" Council, Head & Neck Centre, University College Hospital, 250 Euston Road, London, NW1 2PG, UK; 2 Department of Surgery, University College London Medical School, London, UK; 3 Department of Otolaryngology/Head and Neck Surgery, Barnet and Chase Farm Hospitals NHS Trust, London, UK; 4 Unit of Oral & Maxillofacial Surgery, Division of Maxillofacial, Diagnostic, Medical and Surgical Sciences, UCL Eastman Dental Institute, London, UK; 5 Center for Optical Diagnostics and Therapy, Erasmus University Medical Center, Rotterdam, the Netherlands; 6 The Beckman Laser Institute and Medical Clinic, The University of California Irvine, Irvine, CA, USA; 7 Department of Pathology, The University of Texas M.D. Anderson Cancer Center, Houston, Texas, USA; 8 Department of Otorhinolaryngology, Plastic Head and Neck Surgery, Aachen University Hospital, RWTH Aachen, Germany; 9 Department of Histopathology, Imperial College and The Hammersmith Hospitals, London, UK; 10 Department of Oral & Maxilofacial Surgery, University Medical Center Groningen, the Netherlands; 11 Virginia Piper Cancer Institute-Abbott Northwestern Hospital, Minnesota, USA; 12 Department of Head and Neck Surgery, Division of Surgery, The University of Texas M. D. Anderson Cancer Center, Houston, TX, USA; 13 National Medical Laser Centre, University College London, London, UK; 14 Center for Optical Diagnostics and Therapy, Department of Radiation Oncology, Erasmus University Medical Center, Rotterdam, the Netherlands; 15 Department of Otorhinolaryngology, Head & Neck Surgery, Ludwig Maximilian University, Munich, Germany; 16 LIFE Center, University Clinic Munich, Munich, Germany; 17 Research Centre for Automatic Control (CRAN), Nancy-University, UMR CNRS, France; 18 Michelson Diagnostics, 11A Grays Farm Production Village, Grays Farm Road, Orpington, Kent, BR5 3BD, UK; 19 Gloucestershire Hospitals NHS Foundation Trust, Gloucester, UK; 20 Department of Biomedical Engineering, Beckman Laser Institute, University of California, Irvine, USA; 21 Dept. of Radiation Biology, The Norwegian Radium Hospital, Montebello, Norway; 22 Department of Oral & Maxillofacial Surgery, Tata Memorial Hospital, Mumbai, India; 23 Department of Otorhinolaryngology, Head and Neck Surgery, Klinikum Bielefeld, Bielefeld, Germany; 24 Department of Oral & Maxillofacial Surgery, Leeds Dental Institute, Leeds, UK; 25 National University of Ireland, Galway, Ireland; 26 Department of Bioengineering, Rice University, Houston, USA; 27 National Cancer Centre, Singapore 169610, Singapore; 28 University of Sao Paulo, Sao Carlos, SP, Brazil; 29 National Research Council Canada-Institute for Biodiagnostics, Winnipeg, Manitoba, Canada; 30 Division of Oncology, Lund University Hospital, Lund, Sweden; 31 Department of Head & Neck Oncology & Surgery, The Netherlands Cancer Institute - Antoni van Leeuwenhoek Hospital, Amsterdam, the Netherlands; 32 Division of BioPhysics and BioImaging, Ontario Cancer Institute, Ontario, Canada; 33 Department of Medical Biophysics, Faculty of Medicine, University of Toronto, Toronto, Canada; 34 Division of Gastroenterology and Hepatology, Mayo Clinic, Florida, USA; 35 Department of Biomedical Engineering, Electrical & Computer Engineering, Physics, Boston University, Boston, USA; 36 Physics and Astronomy, University of Pennsylvania, Philadelphia, USA

## Abstract

The complete surgical removal of disease is a desirable outcome particularly in oncology. Unfortunately much disease is microscopic and difficult to detect causing a liability to recurrence and worsened overall prognosis with attendant costs in terms of morbidity and mortality. It is hoped that by advances in optical diagnostic technology we could better define our surgical margin and so increase the rate of truly negative margins on the one hand and on the other hand to take out only the necessary amount of tissue and leave more unaffected non-diseased areas so preserving function of vital structures. The task has not been easy but progress is being made as exemplified by the presentations at the 2nd Scientific Meeting of the Head and Neck Optical Diagnostics Society (HNODS) in San Francisco in January 2010. We review the salient advances in the field and propose further directions of investigation.

## Introduction

**Figure 1:**
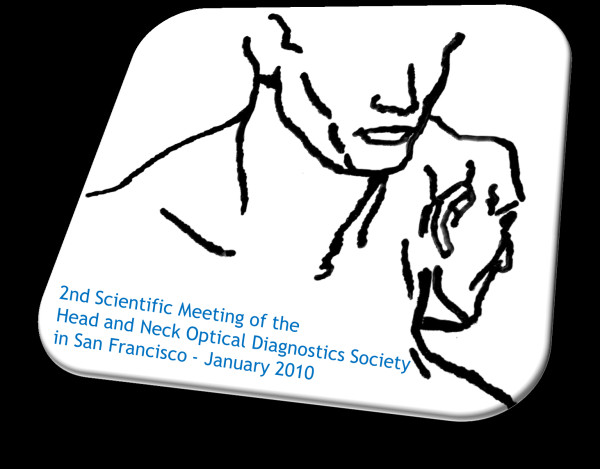
The Head and Neck Optical Diagnostics Society (HNODS) logo

Optical technologies are being used not only to detect disease but also direct treatment (laser) and activate photodynamic therapy as well as in cases aid in the activation of nano-devices to deliver a therapeutic effect. We briefly outline some of the excellent evidence presented in the 2nd Scientific Meeting of the Head and Neck Optical Diagnostics Society (HNODS), (Figure 1) in San Francisco in January 2010.

### I. Diagnostic

#### Disease detection

Optical diagnostics have proved to be a reliable resource that can be used to provide an instant diagnosis of soft and, more recently, hard tissue diseases. In the field of head and neck malignancy, most of the experimental spectroscopy work has been performed using fluorescence spectroscopy, Raman spectroscopy, elastic scattering spectroscopy, micro-endoscopy and optical coherence tomography. These have all shown a marked increase in the sensitivity and specificity when compared to both clinical examination and frozen section analysis [1].

Optical biopsies can be acquired through different modalities; each has it own mechanism of action and requires different modes of data analysis. However, they share the ability of being able to provide a real time, non-invasive and *in situ *optical signature. Most of these techniques have been applied only in clinical trials and are yet to be employed in clinical practice, with the exception of fluorescence spectroscopy. Results from these trials are very promising and current results indicate the possibility of these techniques being applied in clinical practice in the next few years. This could have a great impact on diagnostics, by reducing the histopathology workload, reducing patient's anxiety, and allowing rapid surgical or adjuvant intervention [1]. Further miniaturization has reduced the size of micro-endoscopic multi-photon imaging devices allowing further clinical applications to be developed [2].

#### Applications of optical technologies for diagnosis of lung pathology

Visualizing the respiratory mucosa in pulmonary airways at the sub-cellular level could yield new insights into pathogenesis of many important diseases. However, current imaging modalities to study the respiratory mucosa lack the required resolution to visualize critical sub-cellular detail such as nuclei and respiratory epithelial cilia [3].

Full-field optical coherence microscopy (FFOCM) is an emerging technique capable of providing reflectance images *in situ *with high spatial resolution in all three dimensions. Liu et al. have developed a FFOCM with an axial sectioning thickness of 1 μm and a high transverse resolution of 0.6 μm. Individual epithelial cells and goblet cells, including their sub-cellular morphologies, were seen. Their results demonstrated the potential of FFOCM to provide detailed micro-structural imaging of pulmonary airways without administration of a contrast medium [3].

Investigating the structure and function of pulmonary alveoli *in vivo *is crucial for understanding the normal and diseased lung. In particular, understanding the three-dimensional geometry and relationship of the terminal alveoli to their neighboring alveoli, alveolar ducts and acini during respiration would be a major advance. However, the lung is an inherently difficult organ to image *in vivo *and the peripheral lung has many compounding challenges not limited to its highly scattering micro architecture, large motion artifacts and difficult access through the bronchial tree. Namati et al. used a high-speed high-resolution optical frequency domain imaging (OFDI) system that is endoscopically compatible for future *in vivo *imaging of human alveoli. They showed that OFDI images reveal clear delineation of alveolar septal walls, demonstrating that high-speed three-dimensional visualization of air filled alveoli is feasible [4].

Lung disease involving the alveoli and distal bronchioles are poorly understood and most commonly studied indirectly via lung function tests. Unglert et al. showed that alveolar microstructure could be resolved in three dimensions in images obtained by intra-vital fluorescence microscopy and optical coherence tomography [5].

Furthermore, lung cancer is the leading cause of cancer-related death, and despite recent efforts to reduce the mortality associated with the disease, patient prognosis remains poor with the current 5-year survival rate under 15%. Detection and diagnosis of lesions arising in the bronchial mucosa remains problematic and as a result they are typically well advanced upon discovery.

Suter et al. described their experience in using optical frequency domain imaging (OFDI) to interrogate the bronchial mucosa of patients with the suspicion of lung cancer. During bronchoscopic evaluation, regions of interest suspicious of cancer or precursor lesions were identified and imaged, in addition to regions of normal appearing mucosa. OFDI imaging of the pulmonary airways may enable the early detection of airway features associated with the development of cancer. When used as a screening tool in high-risk patients, it is hoped that early detection of airway-associated cancer with OFDI will result in a decrease in patients' mortality [6].

#### Applications of optical technologies for diagnosis of oral and head and neck pathology

Non-invasive differentiation of pre- and early malignant mucosal changes may be helpful to reduce the morbidity and shorten the time to diagnosis for the patients concerned. Optical Coherence Tomography (OCT) seems to be well suited to reach this goal. Volgger et al. reviewed 82 primary leukoplakic or erythroplakic mucosal lesions of the upper aero-digestive tract (UADT) prospectively using an *in vivo*, time-domain OCT. They found that down to a depth of 1.5 mm, micro-anatomical structures were clearly identifiable on the OCT images. OCT reached a sensitivity of 100% and a specificity of 92% or 75% (investigator unblinded or blinded to visual inspection), respectively. This method seems highly promising for early, non-invasive tumour diagnosis in the UADT [7].

Optical biopsy systems have been investigated for various clinical applications; however the main interest remains in the diagnosis and monitoring of premalignant and malignant conditions. Hamdoon et al. acquired suspicious oral lesions from 120 patients which were then subjected to immediate *ex vivo *Swept-Source Frequency-Domain OCT. They suggested that the use of OCT in clinical examination and monitoring can be invaluable tool for inexperienced clinicians [8]. Optical coherence tomography (OCT) is a promising method in the early diagnosis of oral cavity cancer. Prestin et al. showed that depending on the location within the oral cavity, the epithelium demonstrated a varying thickness. The highest values were found in the region of the tongue and the cheek, whereas the floor of the mouth showed the thinnest epithelium [9].

Rubinstein et al. found that the OCT system is non-invasive and easy to incorporate into the operating room as well as to the outpatient clinic. It requires minimal set-up and requires only one person to operate the system. OCT has the distinctive capability to obtain high-resolution images, where the microanatomy of different sites can be observed. OCT technology has the potential to offer a quick, efficient and reliable imaging method to help the surgeon not only in the operating room but also in the clinical setting to guide surgical biopsies and aid in the decision making of different head and neck pathologies [10].

For oral cancer, biopsy has a low specificity because of a thick keratin layer that often covers potential malignancies. Sterenborg et al. investigated the possibility to distinguishing potentially malignant visible lesions from benign ones using differential pathlength spectroscopy (DPS) to reduce the number of unnecessary biopsies in 75 suspicious lesions and 35 clinically normal locations that were not biopsied. They showed that the area under the ROC curve was 0.951 [11]. Pierce et al. developed a portable system to enable high-resolution evaluation of cellular features within the oral mucosa *in situ*. This system is essentially a wide-field epi-fluorescence microscope coupled to a 1 mm in diameter flexible fiber-optic imaging bundle capable of imaging nuclear size and nuclear-to-cytoplasmic ratio following topical application of a fluorescent labeling solution [12].

The development of lymphadenopathy in the neck has many causes, in children it is often found in relation to infection and in a small but significant number it is the first presentation of lymphoma. In adults neoplastic causes predominate for example, lymphoma, squamous cell carcinoma and adenocarcinoma. The treatment modalities and prognosis for these conditions varies enormously and in the case of squamous cell carcinoma, an excision biopsy can lead to significant morbidity. Orr et al. investigated the ability of Raman spectroscopy to differentiate between the major neoplastic diseases of lymph nodes presenting within the neck. Pre-treatment accurate diagnosis is imperative and is a compelling argument for investment in the development of accurate, sensitive and minimally invasive diagnostic techniques, such as Raman spectroscopy [13].

Thyroid cancer is the most common endocrine malignancy. The standard of care in the management of a patient with a thyroid nodule is fine-needle aspiration (FNA) biopsy with cytological evaluation. While 5-10% of nodules are malignant, 10-25% of FNAs' are indeterminate. Consequently, about twice as many patients undergo surgery for a suspicious lesion that turns out to be benign to patients undergo surgery for a known malignant lesion. A more accurate molecular and ultra-structural based algorithm would be useful to improve diagnostic accuracy. Non-invasive optical tissue diagnosis mediated by fiber-optic probes can be used to perform non-invasive, or minimally-invasive, real-time assessment of tissue pathology *in situ*. Elastic light-scattering spectroscopy (ESS) is a point spectroscopic measurement technique, which is sensitive to cellular and sub-cellular morphological features. Normal and abnormal tissues can generate different spectral signatures as a result of changes in nuclear size, density, and other sub-cellular features, the optical-spectroscopy equivalent of histopathological readings. ESS is optimal for use in the small-volume area as found in thyroid FNA. An important advantage of ESS is that it provides an objective and quantitative assessment of tissue pathology that may not require on-site special expertise and subjective image interpretation as in conventional histopathology. Rosen et al. described their experience in the clinical application of elastic scattering spectroscopy in the thyroid [14].

Within the discipline of Otology, Just et al. used OCT images and 3D reconstructions demonstrate the usefulness of OCT to measure the drilling cavity, to visualize the inner ear structures, and to obtain micro-anatomical information about the round and oval window niche. They suggested that OCT-guided drilling allows identification of the intact inner ear more precisely [15]. Optical coherence tomography is a unique technique to visualize subsurface tissue structures with a resolution below 10 μm during microsurgery without tissue contact. A non-contact volumetric imaging with less than 15 μm resolution can guide microsurgery in Otolaryngology (i.e. middle ear or tumor surgery of the vocal fold) and in other medical disciplines [16].

Visible light is a source of energy known to activate the visual system through absorption by photoreceptors in the eye. When the "stress-confinement" condition is fulfilled, laser light can induce an acoustic signal through an opto-acoustic effect. Wenzel et al. sought to assess if visible light with parameters that induce an optoacoustic effect (i.e., 532 nm, 10 ns pulses) could be used to stimulate the peripheral hearing organ at ear drum and middle ear level. The group demonstrated that visible light can be used to activate the peripheral hearing organ when applied at the ear drum level or on bony structures within the middle ear that can transmit vibrations to the cochlea or inner ear. They suggested that the technique could be used to improve implantable and non-implantable hearing [17].

Wisweh et al. showed that automated classification of laryngeal lesions using optical coherence tomography data can be helpful in making a faster and safer diagnosis. A change in the epithelial layer thickness seems to be an effective indicator for laryngeal cancer and its precursors [18].

Within the discipline of dentistry, Choo-Smith et al. are developing a combined optical coherence tomography (OCT) and polarized Raman spectroscopy (PRS) system for the detection of early non-cavitated dental caries. OCT provides high resolution morphological depth imaging of incipient caries. In combination, OCT and PRS have potential for detecting and monitoring early lesions with high sensitivity and high specificity [19].

#### Guidance of therapies

Optical technologies improve a clinician's ability to discriminate between normal and diseased tissue. This has obvious advantages including automation, objectivity and reproducibility to give consistent results with defined accuracies.

In the treatment of cancer, the fundamental surgical goal is to remove all local malignant disease and leave no residual malignant cells. Studies have demonstrated the benefit of achieving negative resection margins in terms of disease free local recurrence and overall survival. The surgical margins for head & neck cancer may vary widely depending on the site of disease. Assessment of tumour resection margins using optical coherence tomography was performed by Hamdoon et al. in 25 patients with early stage oral squamous cell carcinoma (OSCC) who underwent local resection. The group found that the junctional epithelium (between positive and negative margins) can be identified by gradual change in epithelial thickness and basement membrane organisation (integrity) from the normal to pathological. They also identified changes in tumour positive margins including hyper-keratinisation, breach of the basement membrane and dis-organised epithelial structure. Moreover, the tumour spread pattern could be identified on the majority of the interrogated tissue [20].

#### Tissue remodeling

The use of optical technologies to reshape tissue structures is important in reconstructive and cosmetic surgery. Electromechanical reshaping (EMR) of cartilage is a promising non-invasive technique with potential for broad application in reconstructive surgery. EMR involves applying direct current electrical fields to localized stress regions and then initiating a series of oxidation-reduction reactions, thus effecting a shape change. Previous EMR studies have focused on macroscopic structural measurements of the shape change effect or monitoring of electrical current flow. OCT provides a means to gauge structural changes in the tissue matrix during EMR. Chen et al. described the application of OCT to image the EMR process to improve our understanding of the mechanisms of action involved and potentially facilitate optimization of this process [21].

Furthermore, in 1992, laser-induced stress relaxation in cartilage was identified. This led to the development of non-ablative laser reshaping of cartilage. Laser septochondral correction is non-invasive, bloodless, painless procedure which takes only 10 minutes to complete and can be performed in outpatient settings. The efficacy and safety of this technology can be improved with the feed back control system measuring temperature and stress distribution in the course of laser treatment. Sobol et al. presented recent results of the research and clinical applications of the technology and equipment for laser reshaping of cartilage in the ENT in 120 patients. The positive results were obtained for 95 percent of the patients in two years follow-up [22].

Grafts obtained from peripheral regions of costal cartilage have an inherent tendency to warp over time. Laser irradiation provides a potential method to control the warping process, thus yielding stable grafts for facial reconstructive surgery. Foulad et al. proposed a model for assessing the effect of laser irradiation on the warping process of costal cartilage using optical technology [23].

### II. Therapeutic

The therapeutic use of optical techniques ranges from the direct use of optical wavelengths for delivering treatment to the use of light to specifically activate photo-chemicals to affect tumour lysis with minimal bystander tissue effects. Since many of these technologies are surgically or microscopically directed, the accuracy and resolution of the treatment volume far exceeds that obtained by radiotherapy.

Stepp & Betz showed the feasibility of semi-quantitative endoscopic guided perfusion measurements using ICG-angiographies in 25 patients with free-tissue transfer to the upper aero-digestive tract. The method was shown to be easy to perform with no adverse events. A method for simultaneous ICG-fluorescence and white light imaging was presented. The team suggested that the methodology was suited to situations with questionable flap vasculature Doppler signals, or when flaps are otherwise difficult to monitor [24,25].

#### Direct use of optical technology

Lasers have been used by a number of researchers to close wounds in controlled laboratory tests over the last 15 years. Larson et al. presented their experience using a device to fuse tissue membranes as an alternative to sutures or staples for the coaptation of mucoperichondrial membranes. Coaptation is accomplished through the controlled application of laser heating to induce protein denaturation and subsequent renaturation across the interface. They identified the important parameters involved in fusing biological tissues using radiation from laser sources following computational modeling of the fusion process based on engineering first-principles from heat transfer, fluid dynamics and optics, and from experimental results on a particular tissue system [26].

An interesting application was presented by Fishman et al. Advancements in implantable auditory prostheses now demand preservation of residual auditory function following the surgery. Atraumatic cochleostomy formation is essential to this goal. Clinically reported hearing outcomes in human implantation are still quite variable in this regard. It was suggested that CO_2 _laser operated with a handheld hollow waveguide can consistently produce cochleostomies without damaging the residual auditory function. Fishman et al. demonstrated that for a careful selection of the laser's power, the safety range for the laser is superior to the safety range of drilling. Particularly important is the finding that multiple laser pulses through the same cochleostomy do not further increase the initial compound action potential (CAP) threshold elevation. Moreover, multiple laser pulses at different locations of the cochlea do not further increase the initial CAP threshold elevation observed after the first laser pulse [27].

Eustachian tube dysfunction is very common and is the predominant cause of otitis media with effusion. Negative middle ear pressure generated by Eustachian tube dysfunction can cause deformation of the collagen layer in the ear drum. Kaylie and Miller presented their findings after laser myringoplasty using the Omniguide hand-held flexible fiber CO_2 _laser in 22 patients which showed immediate hearing improvement and eardrum contraction [28].

CO_2 _trans-oral laser microsurgery (TLM) is an emerging technique for the management of laryngeal cancer and other head and neck malignancies. This technique has become more widely used by head and neck surgeons progressively replacing traditional open surgical procedures because it is better at preserving organ function with lower overall morbidity. The CO_2 _laser is coupled to a micromanipulator and microscope, which provides enhanced tumor visualization and the ability to perform precise tissue cuts, obtain excellent haemostasis, and avoid damaging the surrounding tissues and structures that are transected during open surgical procedures. Armstrong et al. reflected upon their decade of experience in TLM, they felt that the improved instrumentation, demonstration of oncologic effectiveness, clinical experience using TLM and decreased morbidity has led to an increased utilization of TLM by head and neck surgeons. Successful surgery requires adequate visualization, precise cutting, controlled depth of tissue penetration, and ability to obtain tissue haemostasis. The full spectrum of laser power settings, spot sizes and energy pulse delivery modes is utilized to resect mucosa, fat, muscle, connective tissue and cartilage while avoiding inadvertent damage to nerves and large vessels, and obtaining adequate haemostasis [29].

The use of CO_2 _laser in the management of oral dysplastic lesions has been put into practice for more than a few years now. The main advantage is the decrease in local tissue morbidity. Very few studies have evaluated recurrence, malignant transformation and overall outcome in patients undergoing such procedure. Jerjes et al. applied CO_2 _laser in a prospective study of 123 oral dysplastic lesions followed-up for 6.4 years. Recurrence and/or malignant transformation of oral dysplasia were observed following laser surgery. They recommended laser resection/ablation for oral dysplasia to prevent not only recurrence and malignant transformation, but also postoperative oral dysfunction encountered by other conventional modalities. The use of trans-oral robotic flexible CO_2 _laser with fluorescence and narrow band imaging control was commended [30].

The incidence of oral squamous cell carcinoma (OSCC) remains high. Oral carcinomas are the sixth most common cancer in the world. Squamous cell carcinoma of the oral cavity has a poor overall prognosis with a high tendency to recur at the primary site and extend to involve the cervical lymph nodes. Jerjes et al. followed-up 73 patients for 3 years in prospective study to evaluate the oncological outcomes following trans-oral CO_2 _laser resection of T_1_/T_2 _N_0 _OSCC. Tumour clearance was primarily achieved in 73 patients. Follow-up resulted in a 3-year survival of 87.8%. Recurrence was identified in 12% of the patients. The mean age of 1^st ^diagnosis of the recurrence group was 76.4 years. Most common oral sites included the lateral border of tongue and floor of mouth. Recurrence was associated with clinical N-stage disease. The surgical margins in this group were also evaluated [31].

Vocal fold scarring can arise from disease or post-surgical wound healing and is one of the predominant causes of voice disorders. Focused ultrafast laser pulses have been demonstrated to create tightly confined sub-surface ablation in a variety of tissue, including vocal folds. Ben-Yakar et al. demonstrated how the unique ability of ultrafast laser ablation to create sub-surface vocal fold microsurgeries could be used for eventually creating a plane in tough sub-epithelial scar tissue into which biomaterials can be injected. They found that the use of relatively high repetition rates, with a small number of overlapping pulses, is critical to achieving ablation in reasonable amounts of time while still avoiding significant heat deposition. Additionally, they used multi-photon fluorescence of the ablation region and SHG imaging of collagen fibers to obtain visual feedback of tissue structure and confirm successful ablation [32].

#### Photodynamic therapy

Analogous to 3D dosimetry planning for radiotherapy attempts are being made to plan interstitial photodynamic therapy (iPDT), using multiple linear light sources positioned within the tumour. In an on-going feasibility study in the Netherlands, 16 patients with incurable SCC at the base tongue have been treated with iPDT as a last treatment option. Preliminary results are encouraging with a long-term complete response in 8 out of 16 patients who have failed standard treatment. There is strong evidence that the partial responders are a direct result of inadequate light delivery [33].

Accurate light dosimetry has not yet been performed during iPDT in the head and neck. van Veen et al. proposed the development of dedicated iPDT verification and planning technology to improve the clinical response and reduce the occurrence of side effects. Their results so far indicate good conservation of functions i.e. swallowing, and excellent local control of the tumour. Interstitial PDT may offer an excellent alternative or adjuvant for conventional treatment modalities [33].

Photodynamic therapy with m-THPC is an established treatment for superficial squamous cell carcinoma and is also being considered for treatment of larger head and neck tumors. Recently, clinical implementation of m-THPC-mediated PDT in the head and neck has not been optimal; a subset of patients has experienced incomplete response. It is well-understood that sufficient quantities of light, drug and oxygen must be present in the targeted tissue in order to deliver sufficient damage. This requirement is complicated by variations in the tissue optical properties and in the photo-sensitizer uptake rates; however, most clinical protocols do not measure the effect of these factors on the PDT dose delivered to individual patients [34,35].

Kanick and de Visscher et al. described the use of optical techniques developed to monitor PDT treatments in pre-clinical models into the clinical treatment of head and neck cancer. Their techniques incorporated reflectance and fluorescence spectroscopic measurements into the PDT-treatment protocol. They found that spectral analysis allowed the extraction of m-THPC concentrations and the quantitative determination of tissue physiological parameters that are important to the PDT-delivered dose (i.e. blood volume and hemoglobin saturation). They described the practical and technical challenges of translating these techniques into the clinical setting [34,35].

Photodynamic therapy, the fourth oncological interventional modality has proved its successfulness in the management of variety of pathologies involving the human body. University College London (UCL) researchers evaluated the outcome following ultrasound-guided iPDT of pathologies involving the head and neck region as well as the upper and lower limbs in 110 patients. Clinical assessment showed that more than half of the patients had "good response" to the treatment and a third reported "moderate response". Radiological assessment comparing imaging 6-week post-PDT to the baseline showed moderate response in half of the patients and significant response in 20% of patients [36].

Optical technologies can be used to activate therapy in a particular location directed by imaging modalities to improve co-location of therapy to disease states. Photodynamic therapy (PDT) is a minimally invasive surgical intervention used in the management of tissue disorders. It can be applied before, or after, any of the conventional modalities, without compromising these treatments or being compromised itself. PDT is valuable for potentially malignant disorders. Hopper et al. on a study of 147 patients showed that 5-ALA-PDT and/or mTHPC-PDT offer an effective alternative treatment for potentially malignant oral disorders. It is associated with excellent functional and cosmetic results and can be used in conjunction with other standard therapies [37].

Shafirstein et al. further showed the safety and efficacy of photodynamic therapy (PDT) in the treatment of oral leukoplakia with 5-aminolevulinic acid (5-ALA) and pulsed dye laser (PDL) confirmed with fluorescence diagnosis system. They determined that high power laser activation allowed completing the laser therapy within 1-3 minutes with a significant response in up to 46% [38].

Professor Biel presented an excellent account of the clinical application of photodynamic therapy for ENT cancers. Carcinoma of the larynx accounts for 25-30% of all carcinomas of the head and neck. Early carcinomas of the larynx (Tis or T1) and severe dysplasia are presently treated with either radiation therapy or surgery alone. Photodynamic therapy has been demonstrated to be effective in the treatment of early carcinomas of the larynx, T_is _and T_1_, with cure rates of 90% with follow-up to 236 months. The advantage of PDT for early carcinomas of the larynx is the ability to preserve normal endo-laryngeal tissue while effectively treating the carcinomas. This results in improved laryngeal function and voice quality. Furthermore, PDT requires a short duration of therapy as compared to radiation therapy. PDT is repeatable and carries less risk than surgical therapy and is performed as an outpatient non-invasive treatment. Importantly, the use of PDT does not preclude the use of radiotherapy or surgery in the future for new primary or recurrent disease [39].

Within oro-pharyngeal oncology the management of base of tongue carcinoma continues to be a major challenge in head and neck oncology especially after chemo-radiation failure and the poor reconstructive prospects of total glossectomy with laryngectomy as the remaining salvage option. Without advances in pathological confirmation of previously irradiated fibrotic tongue base tissue the efficacy of robotic trans-oral laser resection is still unproven but very promising. Jerjes et al. evaluated the outcome following ultrasound-guided interstitial photodynamic therapy (US-iPDT) of stage IV tongue base carcinoma in 33 patients followed-up for 18 months. Their clinical assessment showed that two-thirds of the patients had "good response" to the treatment and a third reported "moderate response" [40].

#### Advances in diagnosis and therapy

Jerjes et al. also described their experiences with a phase I dose escalating study of photochemical internalization (PCI), which is a novel technology that facilitates the delivery of oncotoxic macromolecules into cytoplasm. The initial mechanism and practical application was described by Berg et al. in 1999. The group evaluated the safety and tolerance of the photo-sensitizer (amphinex) that is used to initiate the photochemical internalization process with bleomycin as the chemotherapeutic agent. The most striking finding was the dramatic tumour responses. The starting dose of Amphinex for the study was set at a level not expected to trigger a PCI response, however there appeared to be a localized synergistic effect with photo-activation [41].

It is now known that the presence of biofilms in the upper aero-digestive tract prevents the adequate treatment of infections because the infecting agents remain sequestered in an environment away from circulating or topical agents such as antibiotics. The treatment of these recidivist infections is difficult although the use of selected detergents has shown promise. An alternative treatment option is the use of PDT which was shown to be effective *in vivo *by Rhee et al. using *H. Influenza *biofilms with over 66% resolution of otitis media, one of the most common childhood diseases [42].

Naturally the next step in the utilization of optical technologies is in the shaping and control of nano devices. An innovative use of optical technology in the activation of nano-devices to promote chondrocyte growth across Ti:Sapphire Femto second laser microstructured titanium prosthesis coated with bioactive proteins was shown by Ilgner et al. to improve bio-integration and sound transmission [43].

In summary, we briefly outlined some of the excellent evidence presented in the 2nd Scientific Meeting of the Head and Neck Optical Diagnostics Society (HNODS) in San Francisco initially relating to disease diagnostics, including detection and guidance for biopsies and treatment and then focusing upon the therapeutic use of optical technologies either directly or by activating treatments.

## Competing interests

The authors declare that they have no competing interests.

## Authors' contributions

All authors have contributed to conception and design, drafting the article or revising it critically for important intellectual content and final approval of the version to be published.
